# Impact of Early Medical Treatment for Transgender Youth: Protocol for the Longitudinal, Observational Trans Youth Care Study

**DOI:** 10.2196/14434

**Published:** 2019-07-09

**Authors:** Johanna Olson-Kennedy, Yee-Ming Chan, Robert Garofalo, Norman Spack, Diane Chen, Leslie Clark, Diane Ehrensaft, Marco Hidalgo, Amy Tishelman, Stephen Rosenthal

**Affiliations:** 1 The Center for Transyouth Health and Development Children's Hospital Los Angeles University of Southern California Los Angeles, CA United States; 2 Gender Management Services Boston Children's Hospital Harvard Medical School Boston, MA United States; 3 Center for Gender, Sexuality and HIV Prevention Lurie Children’s Hospital Northwestern Feinberg School of Medicine Chicago, IL United States; 4 The Potocsnak Family Division of Adolescent and Young Adult Medicine Ann & Robert H Lurie Children’s Hospital of Chicago Northwestern University Feinberg School of Medicine Chicago, IL United States; 5 Child and Adolescent Gender Center Benioff Children's Hospital-San Francisco University of California San Francisco San Francisco, CA United States

**Keywords:** transgender youth, gender dysphoria: puberty blockers, gender affirming hormones

## Abstract

**Background:**

Transgender children and adolescents (ie, those who experience incongruence between assigned sex at birth and internal gender identity) are poorly understood and an understudied population in the United States. Since 2008, medical care for transgender youth has generally followed guidelines developed by professional consensus, given the paucity of empirical research, particularly in the US setting.

**Objective:**

The objective of this research was to provide evidence-based data to inform clinical care for transgender youth. The study aims (1) to evaluate the impact of gonadotropin-releasing hormone agonists administered for puberty suppression on mental health, psychological well-being, and metabolic and physiologic parameters including bone health in a cohort of children and adolescents (Tanner stages 2-4) with gender dysphoria, comparing baseline and follow-up assessments, and (2) to determine the impact of gender-affirming hormones (eg, estradiol and testosterone) administered for phenotypic gender transition on mental health, psychological well-being, and metabolic and physiologic parameters in a cohort of adolescents with gender dysphoria, comparing baseline and follow-up assessments.

**Methods:**

The study uses a longitudinal observational design to examine the outcomes of existing medical treatment protocols for gender dysphoria in two distinct cohorts: youth initiating puberty suppression and youth pursuing a phenotypic gender transition. Data on routine anthropometric and physiologic parameters are collected through chart abstraction, questionnaires, and research interviews in the 24-month study period. Audio computer-assisted self-interview and individual interview survey instruments are used to collect demographic, mental health, psychosocial, and behavioral data from parents and youth in the blocker cohort and only from youth in the gender-affirming hormone cohort at baseline and 6, 12, 18, and 24 months.

**Results:**

Participant recruitment commenced in July 2016, and enrollment was completed in September 2018. A total of 90 participants were enrolled in the blocker cohort and 301 participants were enrolled in the gender-affirming hormone cohort. Findings based on baseline data are expected to be submitted for publication in 2019.

**Conclusions:**

This longitudinal, observational study is collecting critical data on the existing models of care for transgender youth that have been used in clinical settings for close to a decade, although with limited empirical research to support them. This research is a direct response to the Institute of Medicine report calling for such studies as well as the needs of clinicians and patients. Results from this study have the potential to significantly impact the medical and mental health services provided to transgender youth by making available rigorous scientific evidence on the impact and safety of early treatment based on the sexual development stage. Ultimately, we aim to understand if early medical intervention reduces the health disparities well known to disproportionately affect transgender individuals across their lifespan.

**International Registered Report Identifier (IRRID):**

PRR1-10.2196/14434

## Introduction

### Background

“Transgender” is a broad term used to describe individuals whose gender self-identification or expression transgresses established gender norms associated with their assigned sex at birth. Specifically, it is the state of one’s gender identity not aligning with one’s assigned sex at birth [1]. The identities and behaviors of transgender individuals are socially and medically stigmatized, resulting in a grossly underserved population at high risk for significant morbidity and mortality. Transgender people often experience gender dysphoria resulting from the incongruence between their assigned sex at birth and their gender identity. The Diagnostic and Statistical Manual of Mental Disorders-5 (DSM-5) outlines the criteria for the diagnosis of gender dysphoria in adolescents and adults as the presence of a marked incongruence between one’s experienced/expressed gender and assigned gender, lasting for at least 6 months, as manifested by at least two of the following: (1) a marked incongruence between one’s experienced/expressed gender and primary and/or secondary sex characteristics (or in young adolescents, the anticipated secondary sex characteristics); (2) a strong desire to be rid of one’s primary and/or secondary sex characteristics because of a marked incongruence with one’s experienced/expressed gender (or in young adolescents, a desire to prevent the development of the anticipated secondary sex characteristics); (3) a strong desire for the primary and/or secondary sex characteristics of the other gender (or a gender different from that assigned at birth); (4) a strong desire to be of the other gender (or a gender different from that assigned at birth); (5) a strong desire to be treated as the other gender (or a gender different from that assigned at birth); and (6) a strong conviction that one has the typical feelings and reactions of the other gender (or a gender different from that assigned at birth). Finally, the individual must also experience clinically significant distress or impairment in social, occupational, or other important areas of functioning [2].

Transgender children and adolescents are a poorly understood and a distinctly understudied population in the United States. The limited available data suggest that transgender youth who are gender dysphoric are at an increased risk for anxiety, depression, suicide, and substance use compared to their peers [3-6]. The development of undesired secondary sex characteristics during puberty often intensifies the distress associated with gender incongruence and increases the risk for these conditions. Current clinical practice guidelines aim to decrease gender dysphoria and ameliorate potential negative health outcomes. Pharmacologic treatment recommendations vary depending on the age and developmental stage of youth with gender dysphoria. For youth in the earliest stages of pubertal development (Tanner stages 2-3; Multimedia Appendix 1 [7]), treatment with a gonadotropin-releasing hormone agonist (GnRHa) is recommended to suppress endogenous puberty and avoid the development of undesired secondary sex characteristics. Among adolescents in the later stages of pubertal development (Tanner stages 4-5), treatment with testosterone or estrogen is recommended to induce the desired masculine or feminine features [8,9]. Although these guidelines have informed care at academic and community centers across the United States, they are based on very limited data. Furthermore, there is minimal available data examining the long-term physiologic and metabolic consequences of gender-affirming hormone treatment in youth. This represents a critical gap in knowledge that has significant implications for clinical practice across the United States.

Research about transgender youth has historically focused on the disproportionate morbidity and mortality among transgender individuals in comparison to the population at large. An Institute of Medicine (IOM) report released in May 2011, entitled “The Health of Lesbian, Gay, Bisexual, and Transgender People,” noted that the existing body of scientific evidence documenting health and well-being of transgender individuals is sparse. The report explicitly called for National Institutes of Health–supported research on transgender health needs, including the development of evidence-based data for providing transgender-specific health care to address gender dysphoria and rigorous research aimed at understanding the health implications of hormone use and other transgender-specific issues. In addition, the IOM report called for longitudinal and cohort studies that incorporate a life course perspective to examine the specific experiences of transgender individuals across different chronological ages [10].

### The “Dutch Model”

Over the past 30 years, a team of specialists in the Netherlands at the *Amsterdam Center of Expertise on Gender Dysphoria* observed that transgender individuals who underwent hormonal gender transition at younger ages assimilated more easily into their “new gender” roles because of greater concordance between their physical appearance and gender [11]. Additionally, many transgender youth going through an undesired endogenous puberty experience distress, commonly resulting in negative emotional, academic, and family functioning [11,12]. Gooren’s lifetime follow-up of 3500 adults treated with hormones revealed increased mortality even with the best of care, due to causes that were overwhelmingly psychosocial (suicide, substance abuse, homelessness, etc) [13]. Based on these clinical observations, Dutch clinician-/investigator-initiated early interventions for transgender youth to suppress undesired puberty with GnRHa have historically been used as the primary strategy for suppression of puberty in children experiencing central precocious puberty [14]. Early results from the first 70 gender dysphoric youth undergoing puberty suppression with GnRHa in the Netherlands showed a decrease in behavioral and emotional problems [15].

In 2006, this “Dutch Model” was published outlining this new approach to the care of transgender youth. Adolescents aged 12 years and older with gender dysphoria are administered GnRHa to minimize further development of undesired secondary sexual characteristics. To induce feminization or masculinization, appropriate gender-affirming hormones (estrogen or testosterone, respectively) are added to the regimen when youth reach the age of 16 years. A recent follow-up study from the Dutch team showed alleviation of gender dysphoria and steady improvement of psychological functioning among 55 transgender young adults whose medical gender transition consisted of puberty suppression, followed by gender-affirming hormones and eventually, gender-affirmation surgery [16].

One important limitation of the Dutch model is the chronological age criteria stating that youth with gender dysphoria need to be at least 12 years of age before initiating suppression of puberty and at least 16 years of age before initiating gender-affirming sex-steroid treatment. It is well documented that many children in the United States are already well into their puberty at the age of 12 years [17,18]. In 2013, Biro et al reported that across three major metropolitan areas in the United States, the median age at onset of stage 2 of breast development was 8.8, 9.3, 9.7, and 9.7 years for African American, Hispanic, white non-Hispanic, and Asian participants, respectively [19]. Use of criteria based on chronological age rather than sexual developmental stage decreases early intervention potential and may overlook key windows of opportunity to decrease the risk for negative mental health outcomes. Only a few studies describe the physiological and psychosocial impact of this treatment protocol for puberty suppression in transgender youth [15,16]. There are a handful of studies that have acknowledged the importance of family support in the psychological health and well-being of youth with gender dysphoria [20-22]; however, there is a significant paucity of data examining the experience of parents/caregivers of youth with gender dysphoria. Many youth seeking services for gender dysphoria related to phenotypic transition will access care at or after the age of majority, as they lack parental consent necessary for initiating medical treatment. For youth who are undergoing puberty suppression in early puberty, the chronologic age dictates the necessity of parent/guardian consent and thus provides an opportunity to collect information about their experiences in this study. Finally, the recommendations from the Dutch model are based on data collected from a homogenous population of white, European youth living in relatively supportive environments and are not necessarily generalizable to multiethnic transgender youth in the United States, particularly given the ethnic differences in the timing of pubertal development [19].

### The Endocrine Society Clinical Practice Guidelines

In 2009, using the best available evidence, but largely based on expert opinion, the Endocrine Society incorporated the Dutch model into the clinical guidelines “Endocrine Treatment of Transsexual Persons,” which includes recommendations for the treatment of transgender youth [9]. Using the Dutch model as a springboard, the Endocrine Society recommended starting treatment with GnRHa for puberty suppression based on sexual development (Tanner staging) rather than chronological age. These “developmental stage, not age” guidelines are based on sexual development at the time of entry into care. The first iteration of the guidelines recommended puberty-blocking medications for youth with gender dysphoria at the beginning stages of puberty (Tanner stage 2 or 3), followed by appropriate gender-affirming hormone therapy at around the age of 16 years. The most recent version of the guidelines, published in 2017, outline compelling considerations for earlier gender-affirming hormone initiation, including potential adverse effects on height and bone mineral density and potential harm to mental health (emotional and social isolation) if initiation of secondary sex characteristics is delayed until the person has reached 16 years of age [8]. However, there are only minimal data supporting earlier use of gender-affirming hormones in transgender adolescents. Since the introduction of these guidelines, no data have been reported in the United States on the physiologic and mental health impact, safety, or tolerability of puberty-suppressing medical interventions with GnRHa for transgender youth, particularly children younger than 12 years of age, leaving a gap in evidence for this practice. Furthermore, the impact of GnRHa on the bone health of transgender children, specifically in those younger than 12 years, remains unknown.

For those patients initiating clinical care in later puberty, gender-affirming hormones are prescribed without GnRHa as a monotherapy, a protocol commonly used in both adolescents and adults. Studies in adult transgender populations have reported on the physiologic impact of gender-affirming hormones [13,23,24], but only a handful have detailed the physiologic impact of puberty blockers and/or gender-affirming hormone administration in transgender adolescents [25,26].

Although the Endocrine Society Clinical Practice Guidelines are widely adopted by providers around the United States and worldwide, there are no formal empirical studies of related clinical outcomes in transgender children and adolescents.

### The Network

The study described here began with the creation of a unique network of four university-affiliated gender clinics across the United States. The Network’s first project—the Transyouth Care Study—was designed to recruit two developmental cohorts of multiethnic transgender youth and conduct a multisite, observational study examining the safety of hormonal interventions and the physiological and psychosocial outcomes associated with these treatments. All four sites (ie, Children’s Hospital Los Angeles/University of Southern California, Boston Children’s Hospital/Harvard University, Lurie Children’s Hospital of Chicago/Northwestern University, and the Benioff Children’s Hospital/University of California San Francisco) employ similar models of care that include a multidisciplinary team of medical and mental health professionals and are considered the national leaders in the care of transgender children and adolescents.

*The Center for Transyouth Health and Development at Children’s Hospital Los Angeles* promotes healthy futures for transgender and gender diverse youth by providing mental health and medical services, research, training, and capacity building that are developmentally informed, affirmative, compassionate, and holistic. The Center has been providing services for gender diverse and transgender youth since the 1990s and currently includes 1500 youth in active care. Staffed by professionals in the fields of Adolescent Medicine, psychology, social work, psychiatry, researchers, and peer navigators, the Center provides individualized care plans for each patient.

*The Gender Development Program at Ann & Robert H Lurie Children’s Hospital of Chicago* provides outpatient services aimed at supporting the physical, mental, and social health of patients and their families as youth progress through gender identity development. Their specialists in pediatric and adolescent gender development recognize that when it comes to providing effective care, “no one size fits all.” Therefore, their goal is to keep families most informed of treatment options and to support them with medical and behavioral health care.

*The University of California San Francisco (UCSF) Child and Adolescent Gender Center (CAGC)* serves as the Pediatric/Adolescent clinical arm of the widely recognized UCSF Center of Excellence for Transgender Health. The CAGC, housed in the Division of Pediatric Endocrinology at UCSF Benioff Children’s Hospital, provides multidisciplinary care to gender nonconforming/transgender youth and adolescents, attracting patients from northern California and beyond. Since the first transgender adolescent patient was seen 10 years ago, the CAGC has served over 950 patients, and its research and outreach activities are funded by grants from the National Institutes of Health and the San Francisco Department of Public Health.

## 

Results from these four sites will likely be generalizable to youth with gender dysphoria residing in urban areas, and while all four sites provide services for some families and youth from surrounding rural areas, the majority of participants are from urban areas. Additionally, it is of critical value to acknowledge that participants recruited from specialized gender centers are likely to experience a specific set of circumstances that may positively impact their capacity to cope with some of the challenges of gender dysphoria. For example, youth who are minors must have parental consent to engage in medical interventions related to gender dysphoria. Parental consent carries an implicit level of parental support, a known factor in the mental health outcomes of youth with gender dysphoria. The data gathered from these cohorts will not be generalizable to youths with gender dysphoria who have no access to services, such as those with little or no parental support, precariously housed, in foster care without intervention advocacy, or geographically located in an area without transgender youth services.

### Specific Aims

The aims of this study are as follows:

- To investigate the impact of medical treatments for gender dysphoria in two developmentally distinct and multiethnic cohorts of transgender youth recruited from four academic sites across the United States via a network of Gender Centers dedicated to their care;
- To evaluate the impact of GnRHa administered for puberty suppression on mental health, psychological well-being, and physiologic parameters including bone health and to document the safety of GnRHa in a cohort of children and adolescents with Tanner stages 2-4 and gender dysphoria, comparing baseline and follow-up assessments; and
- To evaluate the impact of gender-affirming hormones administered for phenotypic gender transition on mental health, psychological well-being, and metabolic/physiologic parameters and to document the safety of gender-affirming hormones in a cohort of adolescents with gender dysphoria, comparing baseline and follow-up assessments.

## Methods

### Study Design

This longitudinal, observational multisite study aims to better understand the impact of medical treatments for gender dysphoria in youth who are initiating puberty suppression or pursuing a phenotypic gender transition with gender-affirming hormones. Participants are being studied prospectively over a 24-month period from either the initiation of GnRHa or gender-affirming hormones. Day 0 for this study is the initial administration of GnRHa (blocker cohort) or the initiation of gender-affirming hormones (gender-affirming hormone cohort). The baseline assessments may be up to 3 months prior to GnRHa or gender-affirming hormone initiation.

This study was approved by the Institutional Review Boards at the Ann & Robert H Lurie Children’s Hospital of Chicago, Boston Children’s Hospital, Children’s Hospital Los Angeles, and the University of California San Francisco.

### Study Population and Recruitment

A total of 90 youth and their parents/caretakers/legal guardians were enrolled in the blocker cohort. In the cohort initiating gender-affirming hormones, 301 participants were enrolled. Youth and parents were recruited from patients seeking care (hormonal intervention to either suppress the progression of puberty with GnRHa or begin phenotypic gender transition with gender-affirming hormones) at any of the four sites.

To be considered eligible for enrollment in the *blocker cohort*, youth must have met the following criteria: presence of gender dysphoria as determined by a clinician; Tanner stage 2, 3, or 4 of sexual development; appropriateness to undergo puberty suppression with GnRHa; age of 8-16 years; ability to read and understand English; and receiving or planning to receive clinical services at a study site clinic. The exclusion criteria were prior utilization of GnRHs, precocious puberty (assigned males at birth younger than 9 years or assigned females at birth younger than 8 years), pre-existing osteoporosis, presence of serious psychiatric symptoms (eg, active hallucinations and thought disorder), or appearing visibly distraught (eg, suicidal, homicidal, and exhibiting violent behavior) at the time of consent or the baseline study evaluation.

To be considered eligible for enrollment in the *gender-affirming hormone cohort*, participants must have met all the following criteria: presence of gender dysphoria as determined by a clinician, appropriateness for initiating phenotypic gender transition with gender-affirming hormones by the team, age of 8-20 years, ability to read and understand English, and receiving or planning to receive services at a study site clinic. The exclusion criteria were prior utilization of gender-affirming hormones, previously or currently enrolled in the blocker cohort, presence of serious psychiatric symptoms (eg, active hallucinations and thought disorder), appearing visibly distraught (eg, suicidal, homicidal, or exhibiting violent behavior) at the time of consent or the baseline study evaluation, or intoxicated or under the influence of alcohol or other substances that would impair the ability to provide true informed consent or understand and answer the questions.

Regarding age, the minimum age in the inclusion criteria for the gender-affirming hormone cohort was decreased from 13 years (as stated in the original grant proposal) to 8 years in order to ensure that potential participants who might be eligible for hormones based on their Tanner stage would not be excluded due to age alone. Additionally, considerations were made for youth who were found to have very low bone density in the screening, which occurs with youths initiating blockers. Only 7 youths under the age of 13 years at the time of enrollment were enrolled into the cross-sex hormone cohort.

Participants are provided a US $100 gift card after completion of the baseline, 12-month, and 24-month visits (these are longer visits that include the Mini International Neuropsychiatric Inventory for Children and Adolescents [MINI] diagnostic interview) and a US $50 gift card after completing the 6- and 18-month visits.

### Data Collection and Measures

Audio computer-assisted self-interviewing (ACASI) survey instruments are used to collect demographic, mental health, psychosocial, behavioral, and physiologic data from parents/caretakers and youths in the blocker cohort and youth only in the gender-affirming hormone cohort. Measures that address the study aims are described below. Survey instruments collect data from four domains: demographic, transgender-specific experiences including gender dysphoria, mental health, and additional psychosocial information including quality of life and relationships with parents and peers. These data are collected at baseline and 6, 12, 18, and 24 months. The ACASI takes approximately 1-1.5 hours to complete. Questions pertaining to suicidality are particularly sensitive; therefore, to protect participants experiencing active suicidal ideation, these items are flagged in the ACASI and the study coordinator facilitating the study visit is immediately notified. This provides an opportunity for staff to check in with participants, direct them to onsite mental health professionals for assessment, and determine whether the study visit should be continued or postponed. An additional check-in with participants occurs at the end of each study visit to address any concerns regarding suicidality.

Mental health diagnoses are assessed at baseline and 12 and 24 months by the MINI Kid or the MINI for participants aged 17 years and older [27]. Parent participation in the MINI Kid for the blocker cohort is optional.

For the blocker cohort, the Child Behavior Checklist (CBCL) will also be completed by participants’ parents/caregivers. For the gender-affirming hormone cohort, behavioral and emotional problems will be assessed by the Youth Self-Report (YSR) module of the CBCL at baseline, 12 months, and 24 months. The CBCL and YSR assign scores for internalizing, externalizing, and total problems based on the DSM-IV guidelines [28]. The MINI Kid or MINI and the CBCL or YSR are completed after the ACASI on the same day or during a second visit within 2 weeks of the first research visit. The following constructs were administered:

#### Blocker Cohort

#### 

Specific ACASI measures for parent/caregiver were demographics and their own religiosity/spirituality and stress. Measures for their child were transgender-specific experiences, anxiety, trauma symptoms, autism, suicidality, depression, social relationships, negative affect, general life satisfaction, and stress/self-efficacy (Multimedia Appendices 2 and 3).

#### Gender-Affirming Hormone Cohort

The measures included demographics, religiosity/spirituality, gender dysphoria, mental health and trauma assessments, depression, suicidality, gender minority stress, resilience autism symptoms, suicidality, quality of life, body esteem, body image, parental support, negative affect, psychological well-being, social relationships, stress, self-efficacy, quality of life, life change events, and behavioral risk (Multimedia Appendix 4).

#### Anthropometric and Physiologic Data for Both Cohorts

Data on anthropometric and physiologic parameters will be routinely collected through chart abstraction, questionnaires, and interviews throughout the study period. Items include lab results, height, weight, blood pressure, diagnoses, prescription medications, Tanner stage, physiologic changes, and bone mineral density as well as dietary calcium intake and weight-bearing exercise (blocker cohort only; Multimedia Appendix 5).

### Statistical Analysis

#### Primary Objective: Effects of Hormonal Interventions on Mental Health and Psychological Well-Being

Hypotheses under the primary objective will be tested in each cohort using repeated measures multivariate analysis of variance (MANOVA) to assess the trajectories of continuous mental health outcomes and psychological well-being over time within each cohort. The MANOVA approach will preserve statistical power to detect significant effects among this set of related continuous outcomes without the inflated Type I error rates associated with a series of individual analysis of variance (ANOVA) or regression analyses. The MANOVA analyses will investigate the changes over time in gender dysphoria, depression, anxiety, trauma symptoms, self-injury, suicidality, body esteem, and quality of life. The model will incorporate time (ie, measurement time points: baseline, 6-month, 12-month, 18-month or 24-month) as a within-participants factor. Asserted gender, age, ethnicity, and other sociodemographic variables may additionally be entered as possible covariates (ie, analysis of covariates) to improve statistical power in order to detect significant time effects. However, we do not propose any *a priori* hypotheses about demographic effects on these outcomes, and any demographic variables that do not contribute significantly to the model will be removed from the analysis to preserve power and increase model parsimony.

In keeping with conventional practice, analysis will first proceed with a review of the Box test for the equality of covariance matrices [29]. Violations of this assumption would require the use of the Pillai trace statistic [30], as opposed to the Wilks lambda statistic, to determine multivariate statistical significance. If, as hypothesized, the within-participant time variable demonstrates significant multivariate effects, the follow-up univariate results will be inspected as appropriate. The assumption of sphericity via the Mauchly test [31] will be checked for each measured outcome; if sphericity is violated, the Huyhn-Feldt correction for degrees of freedom will be applied to that outcome [32]. Finally, for outcomes showing significant time effects, linear and quadratic contrasts will be checked for significance, and marginal means will be computed and plotted to create a visual display of significant trajectories. An *a priori P* value of .05 will be applied as the criterion for statistical significance in all analyses.

#### Secondary Objective: Safety of Hormonal Interventions

Unlike the mental health and psychological well-being measures, the question of interest for metabolic and physiological parameters is not whether there are significant fluctuations over time (which may or may not be meaningful), but rather whether initiation of hormonal interventions pushes any physiological indicator into a clinically unsafe range, that is, above or below the safety cutoff values predetermined from previous literature and clinical guidelines. Safety will be assessed cross-sectionally with one-sided one-sample *t* tests comparing cohort mean scores to the cutoff value. We hypothesize that the cohort means will be significantly lower than the cutoff score. We will use the Benjamini-Hochberg procedure to account for inflated family-wise alpha due to multiple comparisons at each time point [33].

Additionally, ranges of raw scores from all patient labs will be computed at each time point as part of the preliminary data cleaning and descriptive analysis phase. This will provide an immediate assessment of whether the indicator value for any individual patient has crossed the safety threshold for that indicator as data are collected at each time point. In the event that any participant experiences an individual increase in laboratory values above or below the threshold, medication adjustments will be made to protect the well-being of the patient according to the discretion of the medical provider at the site where they are receiving care regardless of the whole-cohort significance test results for that time point.

#### Bone Density in the Blocker Cohort and Gender-Affirming Hormone Cohort With Previous Gonadotropin-Releasing Hormone Agonist Experience at Puberty

We will use repeated measures ANOVA to estimate trajectories of raw and age-matched bone density scores over time in the blocker cohort and the gender-affirming cohort who previously utilized GnRHa to delay pubertal development. Gender and sociodemographic variables may be entered as possible covariates, linear and quadratic contrasts will be assessed, and marginal means will be computed and plotted to create a visual display of trajectories for both outcomes. We hypothesize that for raw scores, the linear term will not differ significantly from zero, indicating net stability in bone density over time (ie, no loss of bone density). However, for age-matched z-scores, the linear term may be negative, as youth receiving GnRHa fail to add bone density at a rate comparable to their age-matched peers.

#### Exploratory Objective: Risk Behavior in Youth Initiating Gender-Affirming Hormones

We will conduct an exploratory assessment of sexual risk and substance use behavior in the gender-affirming hormone cohort, using repeated measures MANOVA to model trajectories of these risk behaviors over time. Gender and sociodemographic variables may be entered as possible covariates. Given that sexual risk and substance use behaviors increase during adolescence in normative samples, we do not specify *a priori* hypotheses regarding the impact of hormone treatment on these risk behaviors in our transgender population; however, linear and quadratic contrasts will be assessed. Significant positive terms (indicating increased risk over time) would be indicative of a typical adolescent risk trajectory, whereas significant negative terms (indicating decreasing engagement in risky behaviors) or nonsignificant time effects (suggesting no net change in risk) would instead support a “treatment-as-prevention” explanation. Again, the Box test will be reviewed for equality of covariance matrices [29], and the multivariate test statistic will be determined accordingly. Sphericity will be assessed via the Mauchly test with the Huyhn-Feldt correction applied as needed [31,32].

#### Additional Analytic Considerations: Site Clustering Effects

Although the observational study is conducted at four sites nationwide, we do not anticipate substantial site effects. To verify this, a group identifier for each participant will be included in the merged analytic dataset, and the intraclass correlation for each outcome will be calculated before conducting multivariate analyses. If, as anticipated, no significant variance is carried at the group level, we will reduce the model to a traditional one-level model. If significant group-level variance emerges, dummy codes to control site-specific variance will be used to enhance statistical power.

## Results

At the close of study enrollment in September 2018, 301 participants were enrolled from all four sites in the gender-affirming hormone cohort (125% of the target) and 90 participants and a parent/caregiver/legal guardian were enrolled in the puberty blocker cohort (102% of the target). We are in the process of conducting the 6, 12, 18, and 24-month visits with the study participants. To date, our follow-up retention rates among participants eligible for each study visit are as follows: 91% for the 6-month visit, 87% for the 12-month visit, 81% for the 18-month visit, and 88% for the 24-month visit.

Initial data from youth enrolled in the blocker cohort across all study sites (n=90) demonstrate that the age of participants ranges from 8 to 16 years at enrollment, with a mean age of 11 (SD 1.5) years. Slightly more than half (51%) of all blocker cohort participants were assigned male at birth, and 49% were assigned female at birth. In addition, 78% of blocker cohort participants self-identified as white individuals; 20%, as Hispanic or Latino individuals; 13%, as multirace individuals; and 3%, as black/African American individuals. While the majority identified as white individuals, this sample is significantly more ethnically diverse than previous cohorts described undergoing puberty suppression. All blocker cohort participants report current enrollment in some form of schooling, ranging from 3rd grade to 11th grade.

Participants in the gender-affirming hormone cohort range in age from 11 to 20 years at enrollment, with a mean age of 16 (SD 1.9) years. About two-thirds of gender-affirming hormone participants (67%) were assigned female at birth and 33% were assigned male at birth. Over half (63%) of gender-affirming hormone participants self-identified as white; 22%, as Hispanic or Latino; 5%, as Asian; 4%, as black/African American; 1%, as American Indian or Alaska Native; and 3%, as multirace. The majority (91%) of gender-affirming hormone participants reported that they were students, with approximately 16% in 8th or lower grades, and 65% were currently in high school. As the first enrolled participants did not complete their year 2 visit until July 2018, no impact data are available to report on the study objectives, aims, and hypotheses.

## Discussion

The lack of data supporting medical interventions for transgender youth, combined with a shortage of providers knowledgeable of the complex psychosocial risk factors facing these young people, contributes to a health disparity and public health crisis of considerable magnitude.

This longitudinal, observational study is collecting critical data on the existing models of care for transgender youth that have been commonly used in clinical settings for close to a decade, although with very limited empirical research to support them. The gap in existing knowledge about the impact of these practices leaves providers and caretakers uncertain about moving forward with the recommended medical interventions for transgender youth seeking phenotypic transition. This research is a direct response to the IOM report calling for such studies as well as the needs of clinicians and patients. Results from this study have the potential to significantly impact the medical and mental health services provided to transgender youth in the United States by making available rigorous scientific evidence outlining the impact and safety of early treatment based on sexual development stage. Additionally, data from this study will help in understanding the impact of the recommended treatment protocols among a diverse, multiethnic cohort of transgender youth more representative of the US population.

The findings from this research have the capacity to substantially expand treatment across the country by providing rigorous evidence to demonstrate the benefits of early treatment and to ultimately decrease the existing health disparities for transgender youth.

Future studies should focus on sexual health (including HIV prevention and treatment for transgender youth), the developmental trajectories and medical needs of nonbinary youth, and long-term potential health risks that may arise over the lifespan from early interventions. Finally, the long-term follow-up regarding attributes including adjustment, satisfaction, happiness, educational, and employment attainment and function are critical to understanding the benefit of early access to medical care for these vulnerable youth.

## Appendix

Multimedia Appendix 1 Tanner stages.

Multimedia Appendix 2 Blocker cohort - youth survey measures.

Multimedia Appendix 3 Blocker cohort - parent survey measures.

Multimedia Appendix 4 Gender-affirming hormone cohort survey measures.

Multimedia Appendix 5 Anthropometric and physiologic data for both cohorts.

## Abbreviations

ACASI: audio computer-assisted self-interviewing
ANOVA: analysis of variance
CAGC: Child and Adolescent Gender Center
CBCL: Child Behavior Checklist
DSM: Diagnostic and Statistical Manual of Mental Disorders
GeMS: Gender Management Service
GnRH: gonadotropin-releasing hormone
GnRHa: gonadotropin-releasing hormone agonist
MANOVA: multivariate analysis of variance
UCSF: University of California San Francisco
YSR: Youth Self-Report

## References

[1] Grossman AH, D'Augelli AR (2007). Transgender youth and life-threatening behaviors. Suicide Life Threat Behav.

[2] (2019). Diagnostic and statistical manual of mental disorders - fifth edition (DSM-5).

[3] Corliss HL, Belzer M, Forbes C, Wilson EC (2007). An evaluation of service utilization among male to female transgender youth: qualitative study of a clinic-based sample. J LGBT Health Res.

[4] Wilson EC, Garofalo R, Harris RD, Herrick A, Martinez M, Martinez J, Belzer M, Transgender Advisory Committeethe Adolescent Medicine Trials Network for HIV/AIDS Interventions (2009). Transgender female youth and sex work: HIV risk and a comparison of life factors related to engagement in sex work. AIDS Behav.

[5] Reisner SL, Vetters R, Leclerc M, Zaslow S, Wolfrum S, Shumer D, Mimiaga MJ (2015). Mental health of transgender youth in care at an adolescent urban community health center: a matched retrospective cohort study. J Adolesc Health.

[6] Olson J, Schrager SM, Belzer M, Simons LK, Clark LF (2015). Baseline Physiologic and Psychosocial Characteristics of Transgender Youth Seeking Care for Gender Dysphoria. J Adolesc Health.

[7] Carswell J M, Stafford DEJ, Neinstein L, Katzman D, Callahan T (2016). Normal Physical Growth and Development. Neinstein’s Adolescent And Young Adult Health Care: A Practical Guide (Adolescent Health Care A Practical Guide).

[8] Hembree W, Cohen-Kettenis Peggy T, Gooren Louis, Hannema Sabine E, Meyer Walter J, Murad M Hassan, Rosenthal Stephen M, Safer Joshua D, Tangpricha Vin, T'Sjoen Guy G (2017). Endocrine Treatment of Gender-Dysphoric/Gender-Incongruent Persons: An Endocrine Society Clinical Practice Guideline. J Clin Endocrinol Metab.

[9] Hembree WC, Cohen-Kettenis P, Delemarre-van de Waal HA, Gooren LJ, Meyer WJ, Spack NP, Tangpricha V, Montori VM, Endocrine Society (2009). Endocrine treatment of transsexual persons: an Endocrine Society clinical practice guideline. J Clin Endocrinol Metab.

[10] (2011). Bisexual, and Transgender Health Issues and Research Gaps and Opportunities. The Health Of Lesbian, Gay, Bisexual, And Transgender People: Building A Foundation For Better Understanding.

[11] Kreukels B, Cohen-Kettenis Peggy T (2011). Puberty suppression in gender identity disorder: the Amsterdam experience. Nat Rev Endocrinol.

[12] de Vries Annelou L C, Cohen-Kettenis Peggy T (2012). Clinical management of gender dysphoria in children and adolescents: the Dutch approach. J Homosex.

[13] Asscheman H, Giltay Erik J, Megens Jos A J, de Ronde W Pim, van Trotsenburg Michael A A, Gooren Louis J G (2011). A long-term follow-up study of mortality in transsexuals receiving treatment with cross-sex hormones. Eur J Endocrinol.

[14] Mul D, Hughes I A (2008). The use of GnRH agonists in precocious puberty. Eur J Endocrinol.

[15] Delemarre-van de Waal H A, Cohen-Kettenis P T (2006). Clinical management of gender identity disorder in adolescents: a protocol on psychological and paediatric endocrinology aspects. European Journal of Endocrinology.

[16] de Vries Annelou L C, McGuire Jenifer K, Steensma Thomas D, Wagenaar Eva C F, Doreleijers Theo A H, Cohen-Kettenis Peggy T (2014). Young adult psychological outcome after puberty suppression and gender reassignment. Pediatrics.

[17] Kaplowitz P, Oberfield S E (1999). Reexamination of the age limit for defining when puberty is precocious in girls in the United States: implications for evaluation and treatment. Drug and Therapeutics and Executive Committees of the Lawson Wilkins Pediatric Endocrine Society. Pediatrics.

[18] Sun S, Schubert Christine M, Chumlea William Cameron, Roche Alex F, Kulin Howard E, Lee Peter A, Himes John H, Ryan Alan S (2002). National estimates of the timing of sexual maturation and racial differences among US children. Pediatrics.

[19] Biro F, Greenspan Louise C, Galvez Maida P, Pinney Susan M, Teitelbaum Susan, Windham Gayle C, Deardorff Julianna, Herrick Robert L, Succop Paul A, Hiatt Robert A, Kushi Lawrence H, Wolff Mary S (2013). Onset of breast development in a longitudinal cohort. Pediatrics.

[20] Olson K, Durwood Lily, DeMeules Madeleine, McLaughlin Katie A (2016). Mental Health of Transgender Children Who Are Supported in Their Identities. Pediatrics.

[21] Simons L, Schrager Sheree M, Clark Leslie F, Belzer Marvin, Olson Johanna (2013). Parental support and mental health among transgender adolescents. J Adolesc Health.

[22] Wilson E, Chen Yea-Hung, Arayasirikul Sean, Raymond H Fisher, McFarland Willi (2016). The Impact of Discrimination on the Mental Health of Trans*Female Youth and the Protective Effect of Parental Support. AIDS Behav.

[23] Futterweit W (1998). Endocrine therapy of transsexualism and potential complications of long-term treatment. Arch Sex Behav.

[24] van Kesteren P, Lips P, Deville W, Popp-Snijders C, Asscheman H, Megens J, Gooren L (1996). The effect of one-year cross-sex hormonal treatment on bone metabolism and serum insulin-like growth factor-1 in transsexuals. J Clin Endocrinol Metab.

[25] Klink D, Caris Martine, Heijboer Annemieke, van Trotsenburg Michael, Rotteveel Joost (2015). Bone mass in young adulthood following gonadotropin-releasing hormone analog treatment and cross-sex hormone treatment in adolescents with gender dysphoria. J Clin Endocrinol Metab.

[26] Vlot M, Klink Daniel T, den Heijer Martin, Blankenstein Marinus A, Rotteveel Joost, Heijboer Annemieke C (2017). Effect of pubertal suppression and cross-sex hormone therapy on bone turnover markers and bone mineral apparent density (BMAD) in transgender adolescents. Bone.

[27] Sheehan DV, Sheehan KH, Shytle RD, Janavs J, Bannon Y, Rogers JE, Milo KM, Stock SL, Wilkinson B (2010). Reliability and validity of the Mini International Neuropsychiatric Interview for Children and Adolescents (MINI-KID). J Clin Psychiatry.

[28] Achenbach T, Ruffle T M (2000). The Child Behavior Checklist and related forms for assessing behavioral/emotional problems and competencies. Pediatr Rev.

[29] Box G (1949). A general distribution theory for a class of likelihood criteria. Biometrika.

[30] Pillai KCS (1955). Some New Test Criteria in Multivariate Analysis. Ann Math Statist.

[31] Mauchly JW (1940). Significance Test for Sphericity of a Normal n-Variate Distribution. Ann Math Statis.

[32] Huynh H, Feldt Ls (1970). Conditions under Which Mean Square Ratios in Repeated Measurements Designs Have Exact F-Distributions. Journal of the American Statistical Association.

[33] Benjamini Y, Hochberg Y (1995). Controlling the False Discovery Rate: A Practical and Powerful Approach to Multiple Testing. Journal of the Royal Statistical Society.

