# Supplementary material for: Impact of Early Medical Treatment for Transgender Youth: Protocol for the Longitudinal, Observational Trans Youth Care Study
Source: JMIR Res Protoc. 2019 Jul 9;8(7):e14434. doi: 10.2196/14434 (PMC6647755; doi:10.2196/14434)
Supplement: Multimedia Appendix 1 [file resprot_v8i7e14434_app1.pdf]

Stage	Pubic Hair	Breast	Testicular volume
I	No pubic hair	No glandular tissue, only the nipple papilla is elevated beyond the flat chest contour	< 4mL
II	Small amount of long, downy hair; may be pigmented	Breast bud forms with minimal glandular tissue, areola widens	4-8 mL
III	Darker, more curly and rougher texture	Breast tissue beyond the nipple areolar complex (NAC border), forming the shape of the breasts	10-15 mL
IV	Adult hair quality in limited area	Increased breast size and differentiation of NAC above the contour of the breast	15-20 mL
V	Adult hair quality that extends to the medial thighs in the shape of an inverted triangle	Adult final size, areola regresses and matches the contour of the breast	>25mL
