# Supplementary material for: Impact of Early Medical Treatment for Transgender Youth: Protocol for the Longitudinal, Observational Trans Youth Care Study
Source: JMIR Res Protoc. 2019 Jul 9;8(7):e14434. doi: 10.2196/14434 (PMC6647755; doi:10.2196/14434)
Supplement: Multimedia Appendix 2 [file resprot_v8i7e14434_app2.pdf]

Blocker Cohort – Youth Survey Measures	
Construct	**Measure**
Time of Completion: Baseline, 6-month, 12-month, 18-month, & 24-month follow-up periods	
Weight Bearing Exercise	Physical Activity Questionnaire
Demographics	Demographic questions for Blocker Cohort Youth
Depression	BDI-Y
Anxiety	Revised Children's Manifest Anxiety Scale: Second Edition (RCMAS-2 – What I Think and Feel)
Quality of Life	Pediatric Quality of Life Inventory – Child Report (PedsQL–CH)
Suicidality	Suicidal Ideation Scale
Self-Harm	Two questions embedded in demographics section
Body Esteem	Body Esteem Scale
Social Relationships	Emotional Support / Friendship / Loneliness / Perceived Hostility / Perceived Rejection – NIH Toolbox
Self-Efficacy	Self-Efficacy (CAT 8-12)– NIH Toolbox
Perceived Parent Support	Parental Support Scale – Youth Version
Resiliency	Connor-Davidson Resilience Scale
Self-Perception	Harter's Self-Perception Profiles for Adolescents & Children
Time of Completion: 6-month, 12-month, 18-month & 24-month follow-up periods	
Side Effects of GnRH Agonists	Physical and emotional effects of hormone blocker use
Time of Completion: 12-month & 24-month follow-up periods	
Adolescent Life-Change Event Scale	Life changes in past 6 months (only participants 9 years and older)

MINI	
Construct	**Measure**
Time of Completion: Baseline, 12-month, & 24-month follow-up periods	
DSM Diagnoses	Mini International Neuropsychiatric Interview for Children and Adolescents – M.I.N.I. Kid (Modules: Major Depressive Episode / Manic Episode / Hypomanic Episode / Panic Disorder / Agoraphobia / Separation Anxiety Disorder / Social Anxiety Disorder (Social Phobia) / Specific Phobia / Obsessive-Compulsive Disorder / Posttraumatic Stress Disorder / Tourette's Disorder / ADHD / Conduct Disorder / Oppositional Defiant Disorder / Anorexia Nervosa / Bulimia Nervosa / Binge-Eating Disorder / Generalized Anxiety Disorder / Adjustment Disorders)
