# Supplementary material for: Impact of Early Medical Treatment for Transgender Youth: Protocol for the Longitudinal, Observational Trans Youth Care Study
Source: JMIR Res Protoc. 2019 Jul 9;8(7):e14434. doi: 10.2196/14434 (PMC6647755; doi:10.2196/14434)
Supplement: Multimedia Appendix 3 [file resprot_v8i7e14434_app3.pdf]

Blocker Cohort – Parent Survey Measures	
Construct	**Measure**
Time of Completion: Baseline, 6-month, 12-month, 18-month, & 24-month follow-up periods	
Demographics	Demographic questions for Blocker Cohort Parents
Service Utilization	Service Utilization Questions
Socio-Economic Status	Socioeconomic Status Questions (for Adults)
Religiosity & Spirituality	Modified Duke University Religion Index (DUREL)
Calcium Intake	Daily Calcium Intake Form
Gender Identity	Parent Report Gender Identity Questionnaire (GIQC)
Social Transitioning	Social Transitioning Scale
Gender Dysphoria	DSM 5 – Chicago adapted
Quality of Life	Pediatric Quality of Life Inventory - Parent Report (PedsQL – PC)
Suicide Attempts	Suicidality Questions
Self-harm	Embedded in demographics questions
Parent Distress/Stress	Parenting Stress Index
Social Relationships	Empathic Behaviors / Peer Rejection / Positive Peer Interactions / Social Withdrawal (Parent Report) – NIH Toolbox
Negative Affect	Anger / Fear / Sadness (Parent Report) – NIH Toolbox
Psychological well-being	General Life Satisfaction / Positive Affect (Parent Report)– NIH Toolbox
Self-Efficacy	Self-Efficacy (Parent Report) – NIH Toolbox
Perceived Parent Support	Parental Support Scale – Parent Version
Autism	Autism-Spectrum Quotient (AQ-10) – Child

Child Behavior Checklist (CBCL) (Collected online through ASEBA system)	
Time of Completion: Baseline, 12-month, & 24-month follow-up periods	
Depression/Externalization	Child Behavior Checklist (CBCL)
DSM Diagnoses [a]	Mini International Neuropsychiatric Interview for Children and Adolescents – M.I.N.I. Kid (Modules: Major Depressive Episode / Manic Episode / Hypomanic Episode / Panic Disorder / Agoraphobia / Separation Anxiety Disorder / Social Anxiety Disorder (Social Phobia) / Specific Phobia / Obsessive-Compulsive Disorder / Posttraumatic Stress Disorder / Tourette's Disorder / ADHD / Conduct Disorder / Oppositional Defiant Disorder / Anorexia Nervosa / Bulimia Nervosa / Binge-Eating Disorder / Generalized Anxiety Disorder / Adjustment Disorders)

[a] Parent/Caretaker participation optional
