# Supplementary material for: Impact of Early Medical Treatment for Transgender Youth: Protocol for the Longitudinal, Observational Trans Youth Care Study
Source: JMIR Res Protoc. 2019 Jul 9;8(7):e14434. doi: 10.2196/14434 (PMC6647755; doi:10.2196/14434)
Supplement: Multimedia Appendix 4 [file resprot_v8i7e14434_app4.pdf]

| Gender Affirming Hormone Cohort Survey Measures | |
|---|---|
| Construct | **Measure** |
| Time of Completion: Baseline, 6-month, 12-month, 18-month, & 24-month follow-up periods | |
| Demographics | Demographic questions for Cross-Sex Hormone Cohort |
| Religiosity & Spirituality | Modified Duke University Religion Index (DUREL) |
| Socio-Economic Status | Socioeconomic Status Questions (for Adolescents & Young Adults) |
| Gender Identity | Transgender Congruence Scale |
| | DSM 5 – Chicago adapted |
| Service Utilization | Dr. Olson's Service Utilization Questions |
| Depression | BDI-II |
| Anxiety | Revised Children's Manifest Anxiety Scale: Second Edition (RCMAS-2 – What I Think and Feel) |
| Quality of Life | Health-Related Quality of Life Scale (modified HIV QOL) |
| Suicidality | Suicidal Ideation Scale |
| Body Esteem | Body Esteem Scale |
| Body Image | Body Image Scale |
| Social Relationships | Emotional Support / Friendship / Loneliness / Perceived Hostility / Perceived Rejection – NIH Toolbox |
| Negative Affect | Anger / Fear / Sadness – NIH Toolbox |
| Psychological Well-being | General Life Satisfaction / Positive Affect – NIH Toolbox |
| Self-Efficacy | Self-Efficacy (CAT 13-17)– NIH Toolbox |
| Perceived Parent Support | Parent Support Scale – Youth Version |
| Resiliency | Gender Minority Stress and Resilience Scale |
| | Connor-Davidson Resilience Scale |
| Sexual Behavior | Sexual Risk Behavior Questions |
| STI History | STI Questions |
| Alcohol/Drug Use | Alcohol, Smoking, and Substance Involvement Screening Test (ASSIST) |
| Autism | Autism-Spectrum Quotient (AQ-10) – Adult |
| History of Blocker Experience | Questions to obtain history of participant's blocker experience |
| Time of Completion: 6-month, 12-month, 18-month & 24-month follow-up periods | |
| Side Effects of Hormone Use | Physical and emotional effects of hormone use for the following hormone treatments: testosterone, progesterone, estrogen, spironolactone and/or other hormone blockers |
| Physical Characteristics | Menstruation (first and last menstrual period); history of chest binding and male chest reconstruction procedures; interest in gender affirming surgeries (for transmasculine participants *only*) |
| Chest dysphoria | Chest Dysphoria Scale (for transmasculine participants *only*) |
| Time of Completion: 12-month, 24-month follow-up periods | |
| Adolescent Life-Change Event Scale | Life changes in past 6 months (only participants 9 years and older) |

| Youth Self-Report (YSR) (Collected Online in ASEBA System) | |
|---|---|
| Construct | **Measure** |

| Time of Completion: Baseline, 12-month, & 24-month follow-up periods | |
|---|---|
| Internalizing/Externalizing | Youth Self-Report (YSR), Child Behavior Check List (CBCL) |

| MINI[a]: | |
|---|---|
| Construct | **Measure** |
| Time of Completion: Baseline, 12-month, & 24-month follow-up periods | |
| DSM Diagnoses | Mini International Neuropsychiatric Interview – M.I.N.I. or M.I.N.I. Kid (Modules: Major Depressive Episode / Manic Episode / Hypomanic Episode / Panic Disorder / Agoraphobia / Social Anxiety Disorder (Social Phobia) / Obsessive-Compulsive Disorder / Posttraumatic Stress Disorder / Anorexia Nervosa / Bulimia Nervosa / Binge –Eating Disorder / Generalized Anxiety Disorder / Antisocial Personality Disorder) |

[a] The M.I.N.I. Kid will be utilized with participants aged 16 and under at the Baseline Visit, and the M.I.N.I. will be utilized with participants17 and older at the Baseline Visit. Refer to table of "Blocker Cohort – Youth Survey Measures" for modules included in M.I.N.I. Kid interview.
