# Supplementary material for: Impact of Early Medical Treatment for Transgender Youth: Protocol for the Longitudinal, Observational Trans Youth Care Study
Source: JMIR Res Protoc. 2019 Jul 9;8(7):e14434. doi: 10.2196/14434 (PMC6647755; doi:10.2196/14434)
Supplement: Multimedia Appendix 5 [file resprot_v8i7e14434_app5.pdf]

Anthropometric and Physiologic Data for Both Cohorts	
Form	**Fields**
Time of Completion: Baseline, 6-month, 12-month, 18-month, & 24-month follow-up periods	
Health Data Report	Height, weight, blood pressure, insurance plan at baseline
Visit Report Log	Study items administered, items administered within window, reasons items not administered or administered outside of window, incomplete items, additional comments
Lab Results	luteinizing hormone, follicle stimulating hormone, estradiol, testosterone, calcium, alkaline, serum phosphorus, vitamin D
Diagnoses log	Diagnosis name, date, and comments
Medications	Medication name, dose, unit, route, frequency, start date, stop date, comments
Breast and Modified Ferriman-Gallwey Measurements (Gender affirming hormone cohort)	Breast hemi-circumference (for transfeminine participants), Modified Ferriman-Gallwey Scale (for transmasculine participants)
Time of Completion: Baseline, 12-month, & 24-month follow-up periods	
QCT/DXA (Blocker)	Read by male/female standards, radiologist read, endocrinologist read, QCT or DXA measurements
